# Moderate Amounts of Vitamin D3 in Supplements are Effective in Raising Serum 25-Hydroxyvitamin D from Low Baseline Levels in Adults: A Systematic Review

**DOI:** 10.3390/nu7042311

**Published:** 2015-04-01

**Authors:** Susan J Whiting, Jean-Philippe Bonjour, Flore Dontot Payen, Brigitte Rousseau

**Affiliations:** 1 College of Pharmacy and Nutrition, University of Saskatchewan, Saskatoon, Saskatchewan S7N 2Z4 Canada; 2 Division of Bone Disease, University Hospitals and Faculty of Medicine, Geneva 14, Switzerland; E-Mail: Jean-Philippe.Bonjour@unige.ch; 3 Yoplait France 92641 Boulogne Billancourt, France; E-Mails: Flore.Dontot@yoplait.fr (F.D.P.); Brigitte.Rousseau@yoplait.fr (B.R.)

**Keywords:** fortification, supplements, 25-hydroxyvitamin D, adults, vitamin D status

## Abstract

There is controversy surrounding the designation of vitamin D adequacy as defined by circulating levels of the metabolite 25-hydroxyvitamin D (25(OH)D). Depending on the cutoff level chosen, dietary intakes of vitamin D may or may not provide sufficient impact upon vitamin D status measured as improvement in serum levels of 25(OH)D. We sought to examine whether modest daily doses (5–20 μg) as found in fortified foods or multivitamin supplements had a measureable impact on vitamin D status, defined as moving from below to above 50 nmol/L, or from less than 30 nmol/L to above 30 nmol/L. Published literature was searched for relevant articles describing randomized controlled trials. Exclusion criteria were: studies not involving humans; review articles; studies lacking blood level data pre- and post-treatment; no control group; bolus treatments (weekly, monthly, yearly); vitamin D <5 μg or >20 μg; baseline 25(OH)D ≥75 nmol/L; subjects not defined as healthy; studies <8 weeks; and age <19 years. Of the 127 studies retrieved, 18 publications with 25 separate comparisons met criteria. The mean rate constant, defined as change in 25(OH)D in nmol/L per μg vitamin D administered, was calculated as 2.19 ± 0.97 nmol/L per μg. There was a significant negative correlation (*r* = −0.65, *p* = 0.0004) between rate constant and administered dose. To determine impact of the dose reflecting the Estimated Average Requirement (EAR) of 10 μg administered in nine studies (10 comparisons), in every case mean 25(OH)D status rose either from “insufficient” (30–50 nmol/L) to “sufficient” (>50 nmol/L) or from “deficient” (<30 nmol/L) to “insufficient” (>30 but <50 nmol/L). Our study shows that when baseline levels of groups were <75 nmol/L, for every microgram of vitamin D provided, 25(OH)D levels can be raised by 2 nmol/L; and further, when groups were deficient or insufficient in vitamin D, there was significant value in providing additional 10 μg per day of vitamin D.

## 1. Introduction 

In the past decade the recognition that vitamin D levels were low in many countries has emerged [1], along with evidence that intakes were suboptimal [2] in the face of situations where skin synthesis of vitamin D was not possible. In 2011, two groups published reference intakes for vitamin D. The Institute of Medicine (IOM) [3] brought forth Recommended Dietary Allowances (RDAs) for vitamin D which were 10 μg (400 IU) for infants, 15 μg (600 IU) for children and adults up to age 70 years, and 20 μg (800 IU) for adults over 70 years. The Endocrine Society [4] published recommendations that were stated as being needed for at-risk groups such as those suffering from bone, kidney, or liver malabsorption problems. These were in the range of 15–25 μg (600–1000 IU) for children and 37.5–50 μg (1500–2000 IU) for adults. In either case, these recommended levels were higher than current dietary intakes of most populations, even those such as Canada and the USA where mandatory and discretionary fortification was already in place [2,5].

To achieve vitamin D adequacy without sun exposure, one needs to ingest vitamin D-containing foods and/or supplements. In countries such as Canada and the USA, there are many in the population taking supplements of vitamin D, either alone or as part of multivitamins; for most of them, vitamin D status (>50 nmol/L) is achievable [6]. The question remains, however, as to whether improvements in intakes of vitamin D should be sought through food fortification or via promotion of dietary supplements. While some societies have released guidelines on safe sun exposure [7], countries at high latitudes cannot depend totally on such a strategy. For fortification or supplementation, the question remains as to what are sufficient levels to make a difference in vitamin D status without exposing the population to intakes exceeding the Upper Level (UL) [8]. The UL for vitamin D was set by the IOM at 100 μg for adults and goes as low as 62.5 μg for ages one to three years [3].

Controversy exists regarding defining vitamin D adequacy. The circulating level of the metabolite 25-hydroxyvitamin D [25(OH)D] is the agreed upon biomarker, but the cut-off level has been debated. In setting RDAs, the Institute of Medicine used 40 nmol/L as the level for population adequacy and 50 nmol/L for individuals, corresponding to intakes of 10 and 15 μg (or 20 μg if >70 years), respectively, for maintaining bone health [3]. The Endocrine Society [4] argued that a threshold of 75 nmol/L is optimal for both bone and non-bone functions, especially in unhealthy individuals, and recommended intakes were higher than those set by the IOM, in amounts up to 37.5 μg (1500 IU) per day. In Europe, Bouillon and other prominent vitamin D scientists have recommended a cutoff for 25(OH)D as 50 nmol/L [9]. Therefore, in terms of population health, modelling fortification requires knowledge of how much intake will improve 25(OH)D levels, especially in those with levels below 50 nmol/L.

Many authors, in the course of publishing results of intervention trials, have provided information on vitamin D status improvement per dose of supplement or fortified food. In 2003, Heaney *et al.* [10] reported that the rate constant for vitamin D dosing (using doses between 25–250 μg) was 0.70 nmol/L per μg , *i.e.*, for every microgram of the increment in vitamin D3 intake per day, serum 25(OH)D3 rose by 0.70 nmol/L at steady state. This initial reporting of an algorithm using the rate constant, to determine the effectiveness of vitamin D supplementation, spurred other authors to report rate constants in their publications. Black *et al.* [11] examined the effect of vitamin D in food fortification, with added amounts ranging between 3 μg and 25 μg, and found an overall rate constant of 1.2 nmol/L increase for each microgram of added vitamin D when studies were combined, however, others disagree with this low estimate of how much dietary vitamin D is needed to form 25(OH)D [12]. Using a systemic review protocol we set out to examine the effect of added vitamin D, whether from supplements or added to food, in doses that more closely represented intakes from current supplements or foods, limited to 20 μg or less. A secondary objective was to examine whether 10 μg made a demonstrable impact on vitamin D status by examining studies of subjects whose baseline 25(OH)D could be defined as inadequate (<50 nmol/L) or deficient (<30 nmol/L).

## 2. Methods

### 2.1. Search Strategy

To identify the pertinent data from randomized controlled trials (RCTs) performed in healthy adult subjects, on the effect of daily vitamin D from 5 μg to 20 μg on 25(OH)D levels, we performed a review of the scientific literature published between 2003 and 2013. Added vitamin D could be from food or supplements. Using Medline, all RCTs and meta-analyses of RCTs published in English or French, and performed in healthy adults were retrieved. Trials performed in subjects aged 0–18 years, or performed in patients suffering from specific diseases such as cancer, diabetes, kidney failure, HIV, were excluded. The words “vitamin D (vitamin D2, vitamin D3, cholecalciferol, ergocalciferol)” “intake” “supplementation” “consumption” were combined with the words “25(OH)D” (and synonyms: 25-hydroxyvitamin D, 25-hydroxy cholecalciferol, calcidiol, calcifediol) and “status/blood/serum/ plasma/concentration”. The keywords were restricted to study titles and abstracts so as to retrieve the most relevant articles. Of the 124 studies retrieved, the following exclusion criteria were applied: studies which tested too high or too low doses of vitamin D (*i.e.*, >20 μg per day or <5 μg per day) or nondaily consumption of vitamin D (e.g., weekly, monthly, yearly administration); studies involving subjects not defined as healthy or having a high baseline 25(OH)D (*i.e.*, >75 nmol/L); studies with non-relevant outcomes, *i.e.*, with no information on the level of vitamin D intakes and/ or on the vitamin D blood status; or studies <8 weeks in length. Of the 25 studies remaining, we examined each for quality using the following criteria: sufficient time (at least 8 weeks in length); using only vitamin D3; and having a control group (which could be placebo-controlled or having no placebo pill) with both baseline and end-line measures of 25(OH)D. In addition, we examined the study of McKenna and Murray [12] for eligible studies and located three further studies that were subsequently included. The final number of studies meeting exclusion and quality criteria was 18, of which 17 were supplement studies [13,14,15,16,17,18,19,20,21,22,23,24,25,26,27,28,29] (three with multiple doses and one providing separate values for men and women) and one study of fortified food [30]. Study design and other parameters for these 18 studies are provided in Table A1. Only studies of vitamin D_3_ met exclusion and quality criteria.

### 2.2. Calculation of Rate Constant and Statistical Analysis

The rate constant represents the amount of vitamin D_3_ converted to 25(OH)D at a specific time after intervention. For both the control (placebo) group and the treatment group, the net change from baseline 25(OH)D of the control (placebo group) is determined by subtraction. The rise (or fall) in 25(OH)D of the control group is subtracted (or added) to the net change of the treatment group. The resulting nmol/L is divided by the dose of vitamin D_3_ administered. For determining associations between dose on rate constant and of baseline 25(OH)D on rate constant, statistical analysis were performed to find Pearson correlation coefficients [31].

## 3. Results

For studies identified in the systematic review, net rise in 25(OH)D was found and rate constants were calculated (Table 1). The net change in 25(OH)D with treatment was calculated using an offsetting factor of net change in the control levels of 25(OH)D when necessary.

**Table 1:** Changes in levels of 25-hydroxyvitamin D in vitamin D intervention studies using small to moderate doses (5–20 μg) in supplements or fortified food.

25(OH)D Measurement (nmol/L)					
Study		Baseline Level ± SD (or CI range) by Dose (μg)	Net rise in 25(OH)D by Dose (μg)		Rate Constant: nmol/L per μg by Dose Level (μg) and Time (month)
Aloia *et al.* 2005 [13]		43.0 ± 16.6 (0)	23.9 (20)		1.20 (20) at 3 months
		48.3 ± 20.9 (20)			
Andersen *et al.* 2008 [14]		M 20.0 (15.0, 25.2) (0)	M 16.0 (10) M 31.8 (20) F 39.4 (10) F 38.4 (20)		M 1.60 (10) M 1.59 (20) F 3.94 (10) F 1.29 (20) at 6 months
		M 22.9 (12.6, 28.2) (10)			
		M 18.9 (13.6, 29.2) (20)			
		F 11.7 (7.5, 19.4) (0)			
		F 10.0 (6.9, 14.3) (10)			
		F 14.0 (8.3, 17.5) (20)			
Bischoff-Ferrari *et al.*2006 [15]		F 63.0 ± 30.3 (0)	F 29 (17.5)		F 1.66 (17.5)
		F 70.0 ± 33.0 (17.5)			At 3 years
Bolton-Smith *et al.* 2007 [16]		57.0 ± 15.2 (0)	22.0(10)		2.2 (10)
		62.5 ± 15.5 (10)			at 12 months
Brazier *et al.* 2005 [17]		17.5 (0)	44.2 (10)		4.42 (10)
		18.25 (10)			at 12 months
Bunout *et al.* 2006 [18]		32.8 ± 6.8 (0)	30.0 (10)		3.0 (10)
		31.0 ± 5.5 (10)			at 9 months
Cashman *et al.* 2009 [19]		58.8 (44,78) (0)	18.6 (5) 32.4(10) 39.0 (15)		3.72 (5)
		51.8 (41, 71) (5)			3.24 (10)
		54.3 (43, 72) (10)			2.6 (15)
		55.1 (40, 70) (15)			at 5 months
Chel *et al.* 2008 [20]		25.2 ± 12.1 (0)	35.9 (15)		2.39 (15)
		23.0 ± 8.3 (15)			at 2 months
Gallagher *et al.* 2012 [21]	37.7 ± 9.1 (0)		32.5 (10) *	3.25 (10)	
	37.8 ± 10.8 (10)			at 12 months	
Islam *et al.* 2010 [22]	35.0 ± 9.4) (0)		31.6 (10)	3.16 (10)	
	37.1 ±12.1 (10)			at 12 months	
Karkkaine *et al.* 2010 [23]	49.2 ± 17.7 (0)		23.7 (20)	1.27 (20	
	50.1 ± 18.8 (20)			at 36 months	
Nelson *et al.* 2009 [24]	61.9 ± 22.6 (0)		25.5 (20)	1.28 (20)	
	62.1 ± 24.0 (20)			at 12 months	
Pfeifer *et al.* 2009 [25]	54 ± 18 (0)		26 (20)	1.3 (20)	
	55 ± 18 (20)			at 12 months	
Pignotti *et al.* 2010 [26]	52.9 ± 21.4 (0)		6.9 (10)	0.69 (10)	
	46.7 ± 14.0 (10)			At 3 months	
Smith *et al.* 2009 [27]	36 ± 17 (0)		15 (10)	1.5 (10)	
	44 ± 18 (10)			at 6 months	
Talwar *et al.* 2007 [28]	43.2 ± 16.8 (0)		29.6 (20)	1.48 (20)	
	46.9 ± 20.6 (20)			at 3 months	
Viljakainen *et al.* 2006 [29]	52.2 ± 19.9 (0)		10.9 (5) 21.4 (10) 35.1 (20)	2.18 (5)	
	46.0 ± 14.3 (5)			2.14 (10)	
	46.5 ± 10.2 (10)			1.76 (20)	
	44.1 ± 13.5 (20)			at 12 weeks	
Bonjour *et al.* 2013 [30]	16.2 ± 0.6 (0)		20.2 (10)	2.02 (10)	
	19.2 ± 1.2 (10)			at 8 weeks	

* as reported by authors.

**Figure 1:**
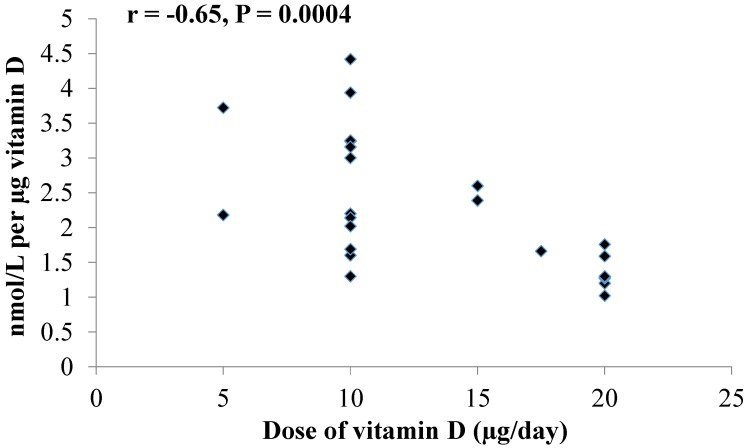
Graph depicts association between rate constant of synthesis of 25-hydroxyvitamin D from its precursor (vitamin D) by dose of vitamin D administered in randomized controlled trials [13,14,15,16,17,18,19,20,21,22,23,24,25,26,27,28,29,30].

The rate constant of change in 25(OH)D expressed as nmol/L per microgram of additional vitamin D was found. These rate constant values were examined two ways. We plotted all rate values by administered dose, and a significant dose response was observed (Figure 1) wherein *r* = −0.65 (*p* = 0.0004). As one study reported a different rate constant depending on baseline level of 25(OH)D [29], we also examined the association between rate constant against starting (baseline) 25(OH)D. When all data were plotted (*n* = 25), there was a negative correlation, though not significant, between baseline 25(OH)D and the change in 25(OH)D per microgram (*r* = −0.28, *p* = 0.175). The average for rate constants for all studies in Table 1, as change in nmol/L per microgram vitamin D provided, was 2.21 ± 0.96 in the 18 studies (25 data points) for an average dose of 13.5 μg (540 IU).

**Figure 2:**
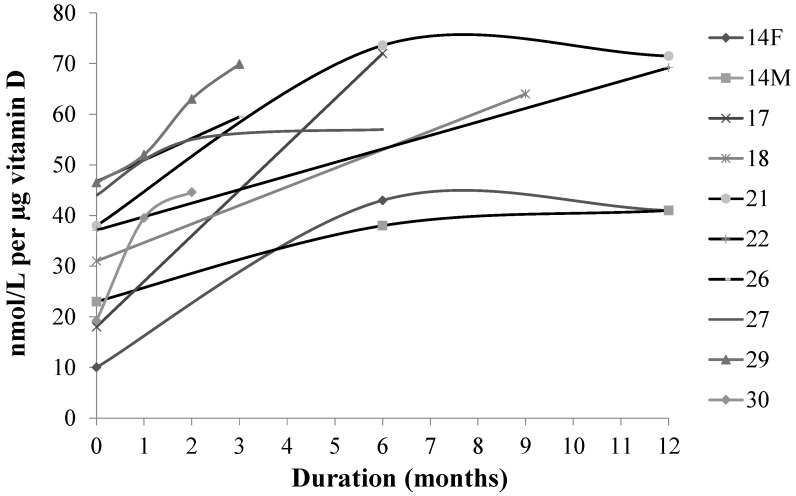
Mean levels of 25-hydroxyvitamin D of treatment groups from randomized controlled trials of 10 μg vitamin D per day. X-axis shows time (months) in study. Dotted line shows 50 nmol/L, which is the cut-off level for sufficiency [3] and the dashed line shows 30 nmol/L, which is the cut-off level for deficiency [3]. Details of the studies [14,16,17,18,21,22,26,27,29,30] are given in Table 1.

Many of the studies shown in Table 1 used a dose of 10 μg, the amount that is the Estimated Average Requirement (EAR) for Canadians and Americans [3]. We used this level to examine whether a moderate dose of vitamin D made an impact on vitamin D status as measured as a rise in 25(OH)D. We excluded two studies that had baseline 25(OH)D over 50 nmol/L and plotted intervention group’s data over time in the study (Figure 2). Our intent in this analysis was to determine whether mean baseline levels of 25(OH)D moved out of the deficient range (<30 nmol/L) into the insufficient range (30–50 nmol/L) or higher, and whether studies where baseline values were in the insufficient range, succeeded in achieving mean levels >50 nmol/L: this was true in every case.

## 4. Discussion

To our knowledge this is the only systematic review of supplemental dosing studies of moderate levels of vitamin D (5–20 μg). Our search found 18 studies that used daily doses of vitamin D in the range of 5 to 20 μg [13,14,15,16,17,18,19,20,21,22,23,24,25,26,27,28,29,30]. For each study, and where applicable, sub-group, the rate constant was found. The rate constant measurement is an indicator of how much added dietary vitamin D, from food or supplement, can raise levels of 25(OH)D over baseline. It is necessary to have studies that are at least two months in length to reach a steady state of 25(OH)D levels [3,10]. Heaney *et al.* [10] concluded that 1 μg increased 25(OH)D by 0.70 nmol/L in a dosing study of >25 μg in subjects with an average baseline level of 70 nmol/L. Using fortification studies only, Black *et al.* calculated the rate constant of fortification studies as being 1.2 nmol/L for every microgram of vitamin D [11]. This would suggest that adding 10 μg to foods would raise 25(OH)D by only 10 nmol/L, a rise that may not change status from deficient (<30 nmol/L) to sufficient (>50 nmol/L). Their analysis was based on foods fortified with 3.5–25 μg/day. Our average rate constant, of 2.21 ± 0.8 is higher, and indicates that adding 10 μg to foods could raise 25(OH)D on average 20 nmol/L.

Our finding of a rate constant closer to 2 nmol/L per microgram is in agreement with the study by McKenna and Murray [12] who reported an average rate constant of 2.1. They averaged values from 41 studies chosen from the Ottawa and Tufts systematic reviews that lasted three or more months. These studies did not include food fortification studies but did include studies of doses as high as 50 μg. One reason their conclusion is similar to ours, despite having a higher cut-off for dose, is their inclusion of studies with vitamin D_2_. Also, their baseline 25(OH)D averaged 39 nmol/L, close to ours (41 nmol/L). Thus, despite differing exclusion criteria, and only a small overlap of studies, a similar conclusion was reached. They did not report, as we have, a significant dose response effect, wherein higher doses produced lower rate constants. This further suggests that when examining impact of vitamin D intake, one must take into account the administered dose.

It has been reported that a lower baseline 25(OH)D should result in a higher rate constant [29]. We found a nonsignificant negative correlation between baseline 25(OH)D and rate constant. The systematic review by Black *et al.* [11] of food fortification studies reported a higher rate constant when baseline 25(OH)D was <50 nmol/L compared to >50 nmol/L. Other factors influencing rate constant derivation include latitude of the study, nature of the assay to determine 25(OH)D, and adherence to treatment [11]; the first two are outlined in Supplemental Table 1, but not analyzed by us for effect. 

The IOM RDA of 15 μg (600 IU) was set to achieve a 25(OH)D level of 50 nmol/L [3]. In Canada, even with its mandatory fortification of milk and margarine, the usual diet supplies only ~5 μg (200 IU) [5]. Canadians who reported taking supplements were more easily able to meet 50 nmol/L levels of 25(OH)D than persons who did not use supplements [6]. Thus, more sources of fortified foods are needed to ensure populations achieve 50 nmol/L and over. We examined the impact of providing 10 μg vitamin D daily to subjects where baseline levels of 25(O)D fell below cutoff levels for either sufficiency (<50 nmol/L) or deficiency (<30 nmol/L) as defined by the IOM’s recent update in vitamin D recommendations [3]. Eleven studies had used this daily dose of vitamin D_3_ [14,16,17,18,19,21,22,26,27,29,30]. When data of treatment groups of those studies where baseline levels were <50 nmol/L were plotted (Figure 2), vitamin D status as defined by cut-off ranges denoting insufficiency and deficiency, improved by one level. From a public health perspective, this suggests adding a total of 10 μg vitamin D3 to the diet, through food fortification, could improve vitamin D status, but only in those needing improvement. 

One concern about fortification has been its potential to cause intakes to exceed the UL for that nutrient [8]. For vitamin D, there is both mandatory and discretionary fortification of foods in the USA [32]. In examining how fortification affects intakes of Americans aged two years and older, Fulgoni *et al.* [33] reported that according to data in NHANES 2003–2006, the intake of vitamin D from both naturally occurring and fortified foods was 4.9 ± 0.1 μg, with none of the population over the ULs that were in use at the time of publication of their findings. However, a large number of highly fortified foods could lead to intakes over the UL.

There are limitations of our study. Studies were very heterogeneous in terms of ages of subjects (from young adults to elderly in institutions). Body mass index was not always provided in studies but in those that did provide this parameter, many used overweight and obese subjects who may have higher vitamin D requirements [34]. As we based the rate constant calculations on these values without regard to BMI, our data do not overestimate a rate constant for a mixed population of overweight and obese subjects. As a limitation, we averaged calculated rate constants without consideration of adjusting for study size (shown in Supplement Table 1). As well, recent work suggests genetic variability is associated with response to vitamin D supplementation and this was not accounted for in any study we examined [35]. Finally, despite much research into vitamin D status, there is now realization that our knowledge of how basal 25(OH)D levels can be achieved is not well understood. Heaney *et al.* [36] calculate that there must be additional food sources of “vitamin D” including 25(OH)D in animal products. These authors, however, also show that sun exposure (cutaneous synthesis) in western countries pays a much less important role, a finding that puts greater justification on supplementation and fortification. 

Overall, our findings suggest the amount of vitamin D added in fortification or through typical multivitamin supplements (10 μg as a daily dose) can have an impact on vitamin D status in those groups at deficient or insufficient levels of. Further, food fortification with levels to satisfy proposed daily values (DVs) on food labels in the USA (20 μg) and Canada (15 μg) is likely to have a meaningful impact. For a food to be considered an “excellent source” of vitamin D at 25 % of the proposed DV in the USA, it must contain 5 μg of vitamin D. Consuming two such foods would improve the status of persons below current cut-off levels for vitamin D sufficiency or deficiency.

## Supplementary Files

Supplementary File 1

## Appendix

**Table A1:** Descriptive and baseline characteristics of vitamin D3 intervention studies.

Design and Population at Baseline							
Study	Dose μg (IU)	Form	Duration (Sampling)	Subjects	Background Intake and Sun Exposure	Assay	*N* per Group
Supplement Studies							
Aloia *et al.* 2005 [13]	20 (800)	Capsule D3 + Ca	3 months	F only, 65–104 years African American BMI	Mean intake 4.6 μg/day	RIA	280-total 104-placebo
Andersen *et al.* 2008 [14]	10 (400) 20 (800)	Tablet D3	1 year (6, 12 months)	F & M 18–64 years; healthy; Pakistani; Denmark BMI ~ 27	Dietary intake averaged 1.7 μg/day	HPLC	199-total 37(F), 27(M)-placebo
Bischoff-Ferrari *et al.* 2006 [15]	17.5 (700)	Tablet D3 + Ca	3 y	≥65 years; healthy; M & F; Boston USA (mixed ethnicity) BMI ~ 26.5	No supplement since at least 2 month ago	CPBA	445-total 125(F), placebo
Bolton-Smith *et al.* 2007 [16]	10 (400)	Tablet D3 + 1000 mg Ca	2 year (12, 24 months)	F only ≥60 years; Caucasian, Scotland BMI ~ 26	No supplement that provided in excess of 10 μg vit D; Vit D intake ~5 μg/day	RIA	244-total 58-placebo
Brazier *et al.* 2005 [17]	10 (400)	Tablet D3 + 500 mg Ca	1 year	F only, >65 years; France	Recruited having 25(OH)D <30 nmol/L; Vit D intake ~2 μg/day	CPBA	191 total 96-placebo
Bunout *et al.* 2006 [18]	10 (400)	Tablets with 800 mg Ca	9 months	F & M, ≥70 years; Chile	Recruited having 25(OH)D <40 nmol/L	Not stated	96-total 46-placebo
Cashman *et al.* 2009 [19]	5 (200) 10 (400) 15 (600)	Capsule D3	22 weeks	F & M; ≥64 years; Caucasian, Ireland	Intervention during winter months	ELISA	225-total 61-placebo
Chel *et al.* 2008 [20]	15 (600)	Tablet D3	4.5 months	F & M 84 ± 6.2 years; nursing home residents; Caucasian, Netherlands	Outside ≤ once/week; no use of vitamin D supplementation; vitamin D fortified food or drink ≤1/day	RIA	338-total 172-placebo
Gallagher *et al.* 2012 [21]	10 (400)	Capsule D3	1 year	F only; 57–90 years; Caucasian Omaha USA	Screened in late winter; Chose low (<50 nmol/L levels)	RIA	41-total 21-placebo
Islam *et al.* 2010 [22]	10 (400)	Tablet D (form assumed to be D3)	1 year	F 18–36 years; Bangladeshi, Bangladesh	Only hands and face uncovered	Enzyme immune- assay	200-total 50-placebo
Karkkaine *et al.* 2010 [23]	20 (800)	Tablet D3	3 years	F only 65–71 years; Finland BMI ~ 27.7	Open-label trial of D + Ca asked all participants to follow usual diet (not specified)	RIA	3139-total 1573-placebo
Nelson *et al.* 2009 [24]	20 (800)	Capsule D3	21 weeks	19–35 years; healthy; F; USA BMI = 25.5	Baseline intake 3.5 μg/day; study run in winter	RIA	112-total 31-placebo
Pfeifer *et al.* 2009 [25]	20 (800)	Tablet D3 + Ca	1 year	F & M; 70–94 years; community-dwelling seniors. Germany and Austria	Vitamin D supplementation was exclusion criterion	RIA	242-total 121-placebo
Pignotti *et al.* 2010 [26]	10 (400)	Tablet D3	3 months	F only 62 ± 8 years; Caucasian Brazil BMI: 26.7	Dietary intake at baseline 3.5 μg/day Vit D supplement use excluded	RIA	64-total 29-placebo
Smith *et al.* 2009 [27]	10 (400)	Tablet D3	5 months	M & F 42 ± 12 years BMI: 19 ± 6	Conducted during winter in Antarctica	RIA	55-total 4(F), 3(M)-placebo
Talwar *et al.* 2007 [28]	20(800)	capsule D3	2 years	F only 59.9 ± 6.2 years; African American, New York USA BMI = 29	Dietary intake : 4.6 μg/day Exclusion of >10 μg/day vitamin D 6 mo before entry	RIA	208-total 104-placebo
Viljakainen *et al.* 2006 [29]	5 (200) 10 (400) 20 (800)	Tablet D3	12 weeks	F only 65–85 years; Helsinki Finland	Dietary intake at baseline ~10 μg/day	HPLC	49-total 12-placebo
Bonjour *et al.* 2013 [30]	10 (400)	Yogurt + 800 mg Ca	8 weeks	F only >60 years; Institutionalized France BMI = 26	Limited sun exposure, winter time, no supplementation	Immuno-Diagnostics System	89-total 27-placebo

* as reported by authors. CPBA = Competitive protein-binding assay.
