# Supplementary material for: Moderate Amounts of Vitamin D3 in Supplements are Effective in Raising Serum 25-Hydroxyvitamin D from Low Baseline Levels in Adults: A Systematic Review
Source: Nutrients. 2015 Apr 1;7(4):2311–23. doi: 10.3390/nu7042311 (PMC4425146; doi:10.3390/nu7042311)
Supplement: Supplementary File 1 [file nutrients-07-02311-s001.docx]

**Supplementary Information**

**Table S1.** Descriptive and baseline characteristics of vitamin D3 intervention studies.

| **Study** | **Design and Population at Baseline** | | | | | | |
| --- | --- | --- | --- | --- | --- | --- | --- |
|  | **Dose μg (IU)** | **Form** | **Duration** | **Subjects** | **Background intake and sun exposure** | **Assay** | **N per group** |
| Supplement Studies | | | | | | | |
| Andersen et al. 2008 [1] | 10 (400) 20 (800) | Tablet D3 | 1 year | F&M 18–64 years; healthy; Pakistani; Denmark BMI ~ 27 | Dietary intake averaged  1.7 μg/day | HPLC | 199-total 37(F),  27(M)- placebo |
| Bischoff-Ferrari et al. 2006 [2] | 17.5 (700) | Tablet D3 + Ca | 3 years | ≥ 65years; healthy; M&F; Boston USA (mixed ethnicity) BMI ~ 26.5 | No supplement since at least  2 mo ago | CPBA | 445-total 125(F), 101(M) –placebo |
| Blum et al. 2008 [3] | 17.5 (700) | Tablet D3+ Ca | 1 year | 65–86 years; F&M; healthy; Boston USA  BMI ~ 27 | Avoided supplements two mo before the trial | CPBA | 257-total 69(F),  56(M)-placebo |
| Bolton-Smith et al. 2007 [4] | 10 (400) | Tablet D3 + 1000 mg Ca | 2 years | F only ≥60 years; Caucasian, Scotland BMI ~ 26 | No supplement that provided in excess of 10μg vit D; Vit D intake ~ 5 μg/day | RIA | 244-total 58-placebo |
| Cashman et al. 2009 [5] | 5 (200)  10 (400)  15 (600) | Capsule D3 | 22 weeks | F&M; ≥64 years; Caucasian, Ireland | Intervention during winter months | ELISA | 225-total 61-placebo |

**Table S1.** *Cont*.

| Chel et al. 2008 [6] | 15 (600) | Tablet D3 | 4.5 months | F&M 84 ± 6.2 years; nursing home residents; Caucasian, Netherlands | Outside ≤ once/wk; no use of vitamin D supplementation; vitamin D fortified food or drink ≤ 1/day | RIA | 338-total  172-placebo |
| --- | --- | --- | --- | --- | --- | --- | --- |
| Gallagher et al. 2012 [7] | 10 (400) | Capsule D3 | 1 year | F only; 57–90 years  Caucasian  Omaha USA | Screened in late winter;  Chose low (< 50 nmol/L levels) | RIA | 41-total 21-placebo |
| Islam et al. 2010 [8] | 10 (400) | Tablet D (form assumed to be D3) | 1 year | F 18–36 years; Bangladeshi, Bangladesh | Only hands and face uncovered | Enzyme  immune- assay | 200-total 50-placebo |
| Karkkaine et al. 2010 [9] | 20 (800) | Tablet D3 | 3 years | F only 65–71 years; Finland BMI ~ 27.7 | Open-label trial of D + Ca asked all participants to follow usual diet (not specified) | RIA | 3139-total  1573-placebo |
| Nelson et al. 2009 [10] | 20 (800) | Capsule D3 | 21 weeks | 19–35 years; healthy; F; USA BMI = 25.5 | Baseline intake 3.5 μg/day; study run in winter | RIA | 112-total 31-placebo |
| Pfeifer et al. 2009 [11] | 20 (800) | Tablet D3 + Ca | 1 year | F&M; 70–94 years;  community-dwelling seniors.  Germany and Austria | Vitamin D supplementation was exclusion criterion | RIA | 242-total 121-placebo |
| Pignotti et al. 2010 [12] | 10 (400) | Tablet D3 | 3 months | F only 62 ± 8 years; Caucasian Brazil BMI: 26.7 | Dietary intake at baseline  3.5 μg/day Vit D supplement use excluded | RIA | 64-total 29-placebo |
| Smith et al. 2009 [13] | 10 (400) | Tablet D3 | 5 months | M&F 42 ± 12 years BMI: 19±6 | Conducted during winter in Antarctica | RIA | 55-total 4(F),  3(M) –placebo |

**Table S1.** *Cont*.

| Talwar et al. 2007 [14] | 20(800) | capsule D3 | 2 years | F only 59.9 ± 6.2 years; African American, New York USA  BMI = 29 | Dietary intake : 4.6 μg/day Exclusion of > 10 μg/day vitamin D 6 mo before entry | RIA | 208-total 104-placebo |
| --- | --- | --- | --- | --- | --- | --- | --- |
| Viljakainen et al. 2006 [15] | 5 (200) 10 (400) 20 (800) | Tablet D3 | 12 weeks | F only 65-85y; Helsinki Finland | Dietary intake at baseline ~  10 μg/day | HPLC | 49-total 12-placebo |
| Food Fortification Study | | | | | | | |
| Bonjour et al. 2013 [16] | 10 (400) | Yogurt + 800mg Ca | 8 weeks | F only > 60 years;  Institutionalized France  BMI = 26 | Limited sun exposure, winter time, no supplementation | Immuno Diag-nostics System | 89-total 27-placebo |

* As reported by authors CPBA = Competitive protein-binding assay

**References**

1. Aloia, J.F.; Talwar S.A.; Pollack, S.; Yeh, J. A randomized controlled trial of vitamin D3 supplementation in African American women. *Arch. Intern. Med.* **2005**, *165*, 1618–1623.
2. Andersen, R.; Molgaard, C.; Skovgaard, L.T.; Brot, C.; Cashman, K.D.; Jakobsen, J.; Lamberg-Allardt, C.; Ovesen, L. Effect of vitamin D supplementation on bone and vitamin D status among Pakistani immigrants in Denmark: A randomised double-blinded placebo-controlled intervention study. *Br. J. Nutr.* **2008**, *100*, 197–207.
3. Bischoff-Ferrari, H.A.; Orav, E.J.; Dawson-Hughes, B. Effect of cholecalciferol plus calcium on falling in ambulatory older men and women: A 3-year randomized controlled trial. *Arch. Inter. Med.* **2006**, *166*, 424–430.
4. Bolton-Smith, C.; McMurdo, M.E.; Paterson, C.R.; Mole, P.A.; Harvey, J.M.; Fenton, S.T.;
   Prynne, C.J.; Mishra, G.D.; Shearer, M.J. Two-year randomized controlled trial of vitamin K1 (phylloquinone) and vitamin D3 plus calcium on the bone health of older women. *J. Bone Min. Res.* **2007**, *22*, 509–519.
5. Brazier, M.; Grados, F. ; Kamel, S.; Mathieu, M.; Morel, A.; Maamer, M.; Sebert, J.L.; Fardellone, P. Clinical and laboratory safety of one year’s use of a combination calcium + vitamin D tablet in ambulatory elderly women with vitamin d insufficiency: Results of a multicenter, randomized, double-blind, placebo-controlled study. *Clin. Ther.* **2005**, *27*, 1885–1893.
6. Bunout, D.; Barrera, G.; Leiva, L.; Gattas, V.; de la Maza, M.; Avendan˜o, M.; Hirsch, S. Effects of vitamin D supplementation and exercise training on physical performance in Chilean vitamin D deficient elderly subjects. *Exp. Gerontol.* **2006**, *41*, 746–752.
7. Cashman, K.D.; Wallace, J.M.; Horigan, G.; Hill, T.R.; Barnes, M.S.; Lucey, A.J.; Bonham, M.P.; Taylor, N.; Duffy, E.M.; Seamans, K.; *et al.* Estimation of the dietary requirement for vitamin D in free-living adults ≥64 y of age. *Am. J. Clin. Nutr.* **2009**, *89*, 1366–1374.
8. Chel, V.; Wijnhoven, H.A.; Smit, J.H.; Ooms, M.; Lips, P. Efficacy of different doses and time intervals of oral vitamin D supplementation with or without calcium in elderly nursing home residents. *Osteoporos. Int.* **2008**, *19*, 663–671.
9. Gallagher, J.C.; Sai, A.; Templin, T.; Smith, L. Dose response to vitamin D supplementation in postmenopausal women: A randomized trial. *Ann. Intern. Med.* **2012**, *156*, 425–437.
10. Islam, M.Z.; Shamim, A.A.; Viljakainen, H.T.; Akhtaruzzaman, M.; Jehan, A.H.; Khan, H.U.;
    al-Arif, F.A.; Lamberg-Allardt, C. Effect of vitamin D, calcium and multiple micronutrient supplementation on vitamin D and bone status in Bangladeshi premenopausal garment factory workers with hypovitaminosis D: A double-blinded, randomised, placebo-controlled 1-year intervention. *Br. J. Nutr.* **2010**, *104*, 241–247.
11. Karkkainen, M.K.; Tuppurainen, M.; Salovaara, K.; Sandini, L.; Rikkonen, T.; Sirola, J.; Honkanen, R.; Arokoski, J.; Alhava, E.; Kroger, H. Does daily vitamin D 800 IU and calcium 1000 mg supplementation decrease the risk of falling in ambulatory women aged 65–71 years? A 3-year randomized population-based trial (OSTPRE-FPS). *Maturitas* **2010**, *65*, 359–365.
12. Nelson, M.L.; Blum, J.M.; Hollis, B.W.; Rosen, C.; Sullivan, S.S. Supplements of 20 μg/d cholecalciferol optimized serum 25-hydroxyvitamin D concentrations in 80% of premenopausal women in winter. *J. Nutr.* **2009**, *139*, 540–546.
13. Pfeifer, M.; Begerow, B.; Minne, H.W.; Suppan, K.; Fahrleitner-Pammer, A.; Dobnig, H. Effects of a long-term vitamin D and calcium supplementation on falls and parameters of muscle function in community-dwelling older individuals. *Osteoporos. Int.* **2009**, *20*, 315–322.
14. Pignotti, G.A.P.; Genaro, P.S.; Pinheiro, M.M.; Szejnfeld, V.L.; Martini, L.A. Is a lower dose of vitamin D supplementation enough to increase 25(OH)D status in a sunny country? *Eur. J. Nutr.* **2010**, *49*, 277–283.
15. Smith, S.A.; Gardner, K.K.; Locke, J.; Zwart, S.R. Vitamin D supplementation during Antarctic winter. *Am. J. Clin. Nutr.* **2009**, *89*, 1092–1098.
16. Talwar, S.A.; Aloia, J.F.; Pollack, S.; Yeh, J.K. Dose response to vitamin D supplementation among postmenopausal African American women. *Am. J. Clin. Nutr.* **2007**, *86*, 1657–1662.

© 2015 by the authors; licensee MDPI, Basel, Switzerland. This article is an open access article distributed under the terms and conditions of the Creative Commons Attribution license (http://creativecommons.org/licenses/by/4.0/).
